# Long-term safety and immunogenicity of the M72/AS01_E_ candidate tuberculosis vaccine in HIV-positive and -negative Indian adults: Results from a phase II randomized controlled trial

**DOI:** 10.1097/MD.0000000000013120

**Published:** 2018-11-09

**Authors:** Nagalingeswaran Kumarasamy, Selvamuthu Poongulali, Faith Esther Beulah, Elaine Jacqueline Akite, Leo Njock Ayuk, Anne Bollaerts, Marie-Ange Demoitié, Erik Jongert, Opokua Ofori-Anyinam, Olivier Van Der Meeren

**Affiliations:** a YRG CARE Medical Centre (YR Gaitonde Centre for AIDS Research and Education), Voluntary Health Services Campus, Taramani, Chennai, India; b GSK, Rixensart and Wavre, Belgium.

**Keywords:** HIV, immunogenicity, M72/AS01_E_, persistence, safety, tuberculosis

## Abstract

**Objectives:**

To assess the long-term safety and immunogenicity of the M72/ Adjuvant System (AS01_E_) candidate tuberculosis (TB) vaccine up to 3 years post-dose 2 (Y3) in human immunodeficiency virus (HIV)-positive (HIV+) and HIV-negative (HIV−) Indian adults.

**Methods:**

This phase II, double-blind, randomised, controlled clinical trial (NCT01262976) was conducted at YRG CARE Medical Centre, in Chennai, India, between January 2011 and June 2015.

Three cohorts (HIV+ participants stable on antiretroviral therapy [ART; HIV+ART+], HIV+ ART-naïve [HIV+ART-], and HIV− participants) were randomised (1:1) to receive 2 doses of M72/AS01_E_ (M72/AS01_E_ groups) or saline (control groups) 1 month apart and were followed up toY3. Latent TB infection was assessed at screening using an interferon-gamma (IFN-γ) release assay (IGRA). Safety and immunogenicity results up to Y1 post-vaccination were reported elsewhere. Here, we report serious adverse events (SAEs), humoral and cell-mediated immune (CMI) responses to M72 recorded at Y2 and Y3.

**Results:**

Of 240 enrolled and vaccinated participants, 214 completed the long-term follow-up part of the study.

In addition to SAEs previously described, between Y1 and Y2 1 M72/AS01_E_ recipient in the HIV+ART+ cohort reported 2 SAEs (sinus cavernous thrombosis and gastroenteritis) that were not considered as causally related to the study vaccine.

Vaccination elicited persistent humoral immune responses against M72. At Y3, seropositivity rates were 97.1%, 66.7%, and 97.3% and geometric mean concentrations (GMCs) were 22.0  ELISA units (EU)/mL, 4.9 EU/mL, and 24.3 EU/mL in the HIV+ART+, HIV+ART-, and HIV− cohorts, respectively. Humoral immune response was lowest in the HIV+ART- cohort.

In M72/AS01_E_ recipients, no notable decrease in the frequency of M72-specific CD4^+^ T-cells expressing ≥2 immune markers among interleukin-2 (IL-2), IFN-γ, tumour necrosis factor alpha (TNF-α) and CD40 ligand (CD40L) was observed at Y3 post-vaccination. Median values (interquartile range) of 0.35% (0.13–0.49), 0.05% (0.01–0.10), and 0.15% (0.09–0.22) were recorded in the HIV+ART+, HIV+ART- and HIV− cohorts, respectively. CD4^+^ T-cell response was lowest in the HIV+ART- cohort.

No CD8^+^ T-cell response was observed.

**Conclusion:**

The cellular and humoral immune responses induced by M72/AS01_E_ in HIV+ and HIV− adults persisted up to Y3 post-vaccination. No safety concerns were raised regarding administration of M72/AS01E to HIV+ adults.

**Clinical Trial Registration:**

NCT01262976 (www.clinicaltrials.gov).

## 1 Introduction

Tuberculosis (TB) is the leading cause of death among people infected with the human immunodeficiency virus (HIV), with 0.4 million deaths reported in 2016.^[1,2]^

The only vaccine currently available against TB, the Bacille Calmette-Guérin (BCG), is given usually in childhood. Although it is effective against severe forms of childhood TB (meningitis and miliary TB), it does not prevent the most prevalent form of TB, pulmonary TB in adults.^[3–5]^ Furthermore, BCG is contraindicated in people with impaired immunity.^[6]^ Therefore, a novel, effective TB vaccine capable of eliciting long-term cellular immunity is urgently needed to target the general susceptible population, including HIV-positive (HIV+) people. Long-term persistence of the vaccine-induced immune responses is essential when targeting the adolescent and young adult population, and should, therefore, be assessed early on in the clinical development of a new vaccine.

The candidate vaccine M72/Adjuvant System (AS01_E_) has been shown to be well tolerated and immunogenic in HIV+ adults receiving antiretroviral therapy (ART),^[7]^ as well as in HIV-negative (HIV−) infants, children and adults.^[8–16]^

We have previously reported results from a phase II randomised trial showing that up to 1 year after vaccination, the M72/AS01_E_ vaccine was well tolerated and immunogenic in ART-stable and ART-naïve HIV+ adults and in HIV− adults living in India.^[17]^ Here, we report the long-term safety and immunogenicity of this vaccine in the same study at 2 and 3 years post-vaccination.

## 2 Methods

### 2.1 Study design and population

This study was a phase II, double-blind (observer-blind), randomised, controlled clinical trial (ClinicalTrials.gov NCT01262976) conducted at YRG CARE Medical Centre, VHS (“YRG CARE”), a tertiary HIV care and research centre in Chennai, India, between January 2011 and June 2015. The protocol was approved by the YRG CARE Institutional Review Board and the Drugs Controller General of India. A summary of the protocol is available at https://www.gsk-clinicalstudyregister.com/ (GSK study ID 113935).

All participants provided written or thumb-printed and witnessed informed consent before any study-specific procedures were undertaken. Good Clinical Practices and the principles of the Helsinki Declaration were followed, and corrective/preventive actions were taken whenever potential or actual issues regarding the conduct of the study conduct were identified or brought to GSK's attention.

**Figure 1:**
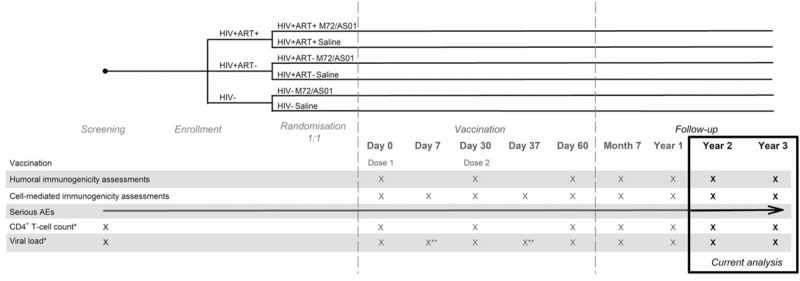
Study design. Day 0 = pre-vaccination; HIV+ART+ = HIV-positive participants on antiretroviral therapy; HIV+ART- = HIV-positive, antiretroviral therapy-naïve participants; HIV− = HIV-negative participants; ^∗^only for HIV+ cohorts; ^∗∗^only for HIV+ART- cohort.

HIV+ participants were recruited from patients registered for follow-up at YRG CARE clinic and were either stable on ART (HIV+ART+ cohort) or ART-naïve (HIV+ART- cohort). HIV− participants (HIV− cohort) were recruited from the general population. Participants in each of the 3 cohorts were randomised (1:1) using an internet-based block randomisation (SASv8.2; SAS Institute Inc.) to receive either 2 doses of M72/AS01_E_ (M72/AS01_E_ groups) or saline (control groups) 1 month apart, and were followed up until 3 years post-dose 2 (Y3) (Fig. 1). To detect latent TB infection, participants were tested at screening with the QuantiFERON TB Gold assay (Cellestis Ltd., Australia), an interferon-gamma (IFN-γ) release assay (IGRA).

Inclusion and exclusion criteria were described in detail in the previous publication.^[17]^

### 2.2 Study vaccine

Each 0.5-mL dose of the M72/AS01_E_ candidate vaccine contained 10 μg of a recombinant fusion protein derived from the *Mycobacterium tuberculosis* antigens Mtb32A and Mtb39A (M72), and the AS01_E_. AS01_E_ contains 25 μg monophosphoryl lipid A, 25 μg *Quillaja saponaria* Molina, fraction 21 (Licensed by GSK from Antigenics LLC, a wholly owned subsidiary of Agenus Inc., a Delaware, USA corporation), and liposome. The control groups received 0.5 mL saline (0.9% NaCl). All injections were administered intramuscularly in the deltoid muscle of the arm.

### 2.3 Study objectives

The primary objective of the study was to evaluate the safety and reactogenicity of M72/AS01_E_ candidate vaccine in adults aged 18 to 59 years with HIV infection.

The secondary objectives were to evaluate the humoral and cellular immunogenicity of M72/AS01_E_.

### 2.4 Safety assessment

Safety and reactogenicity up to 1 year after vaccination (Y1) were assessed as previously described.^[17]^ Serious adverse events (SAEs) were recorded until study end.

For the HIV+ cohorts, CD4^+^ T-cell count and HIV-1 viral RNA load were monitored at screening, day (D) 0, D30, D60, month (M) 7, Y1, Y2, and Y3, and additionally at D7 and D37, only for viral load (in the HIV+ART- cohort) (Fig. 1).

### 2.5 Immunogenicity assessment

Blood samples for the assessment of humoral and cell-mediated immune (CMI) responses were collected at D0, D30, D60, M7, and Y1, Y2, Y3, and additionally at D7 and D37, only for CMI responses (Fig. 1).

M72-specific immunoglobulin G (IgG) antibodies were measured by enzyme-linked immunosorbent assay (ELISA) as previously described.^[17]^ The cut-off for seropositivity was set at 2.8 ELISA units (EU)/mL. For analyses purposes, a titer value of 1.4 EU/mL was given to seronegative participants.

M72-specific CD4^+^ and CD8^+^T-cells expressing at least 1 cytokine among IFN-γ, interleukin 2 (IL-2), tumour necrosis factor alpha (TNF-α) and CD40 ligand (CD40L) were detected by intracellular cytokine staining (ICS) as previously described.^[17]^ Results are presented as background-subtracted frequencies of M72-specific CD4^+^ and CD8^+^ T-cells expressing at least 2 of the above immune markers.

### 2.6 Statistical analysis

Safety analyses were conducted on the total vaccinated cohort (TVC) and immunogenicity analyses on the according-to-protocol (ATP) cohort, as previously described.^[17]^

Anti-M72 seropositivity rates and geometric mean antibody concentrations (GMCs) were calculated with 95% confidence interval (CI). ICS data were expressed as percentages of M72-specific CD4^+^/CD8^+^ T-cells of total CD4^+^/CD8^+^ T-cells. The frequency of CD4^+^ T-cells expressing at least 2 immune markers among IL-2, IFN-γ, TNF-α, and CD40L was computed by summing all the combinations with at least 2 immune markers after having subtracted the number of T-cells for each combination for the background frequencies from the number of T-cells for the stimulated frequencies. For each combination, if the difference was <0, the value of 1 was given.

Comparisons between cohorts were carried out using the Satterthwaite test for humoral immune responses and the Wilcoxon rank-sum test for ICS results. Within a cohort, results for the different timepoints were compared using the paired t-test for humoral immune responses and the Wilcoxon signed rank test for ICS results. The level of significance was set at 0.05 for all statistical tests.

In addition, frequencies of M72-specific CD4^+^T-cells expressing at least 2 immune markers among IL-2, IFN-γ, TNF-α, and CD40L stratified by IGRA status at screening, were also tabulated for all cohorts and timepoints. Comparisons between the cohorts for this analysis should be considered purely exploratory, due to non-adjustment for multiplicity of tests and a potential lack of statistical power.

## 3 Results

### 3.1 Study population

**Figure 2:**
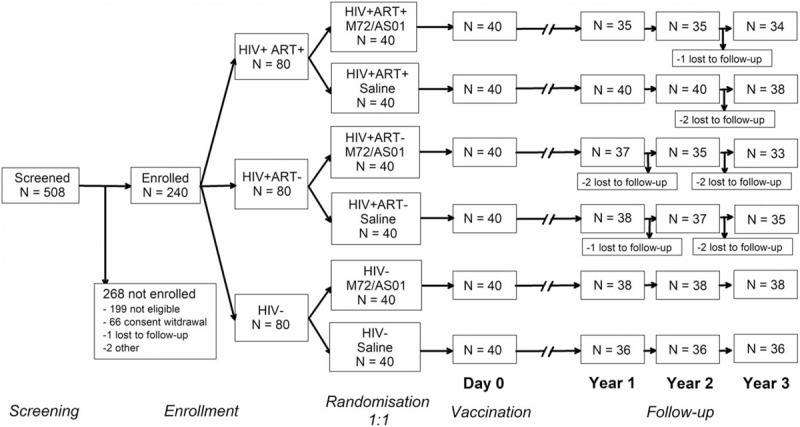
Participant flow. HIV+ART+ = HIV-positive participants on antiretroviral therapy; HIV+ART- = HIV-positive, antiretroviral therapy-naïve participants; HIV− = HIV-negative participants; N =  number of participants.

Of the 240 enrolled and vaccinated participants, 214 completed the long-term follow-up part of the study (Fig. 2). One participant did not consent to the long-term follow-up, and 25 were withdrawn, predominantly because they were lost to follow-up. Two participants from the M72/AS01_E_ group (HIV+ART- cohort) were withdrawn due to an SAE.

Demographic characteristics of the participants have been reported previously.^[17]^ Of the 240 enrolled participants, 72.5% were BCG-vaccinated (had a BCG scar or a history of BCG) and 40.0% were IGRA-positive.

### 3.2 Safety

Safety and reactogenicity results up to 1-year post-vaccination were previously reported.^[17]^

From year 1 to year 3 post-vaccination, 2 SAEs were reported by the same participant, M72/AS01_E_ recipient from the HIV+ART+ cohort. The 44 -year-old participant was diagnosed with cavernous sinus thrombosis, a pre-existing condition observed following a computerised tomography scan performed 425 days post-dose 2 for other causes. The SAE was not resolved at the end of the study. The same participant reported mild gastroenteritis 468 days post-dose 2, of which the participant recovered within 3 days. None of these SAEs were considered to be related to the study vaccine.

There was no observed pattern of changes in CD4^+^ T-cell counts or viral load up to year 3. Both parameters remained comparable between vaccine and control groups throughout the study.

### 3.3 Anti-M72 IgG responses

**Figure 3:**
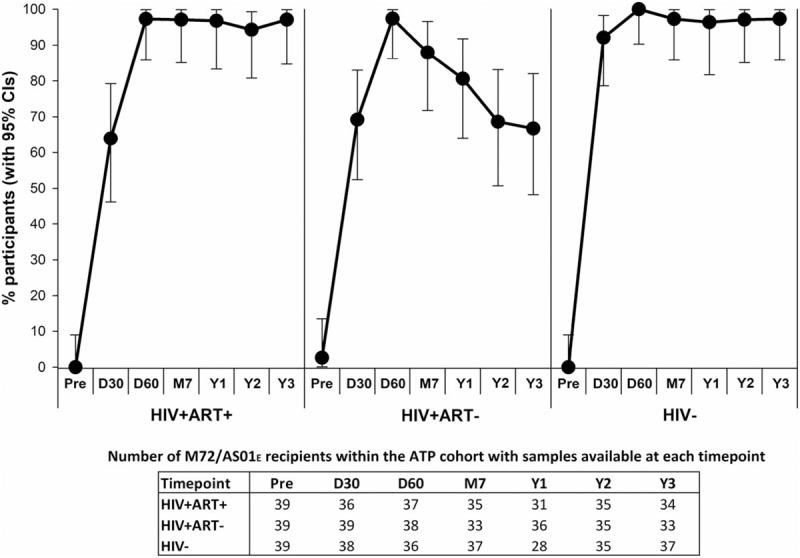
Seropositivity rate. The percentage of participants seropositive for antibodies against M72 at each timepoint, along with 95% CIs, is presented for participants who received M72/AS01_E_ and were included in the ATP cohort for immunogenicity. ATP = according-to-protocol; CI = confidence interval; D30 = 30 days post-dose 1; D60 = 30 days post-dose 2; HIV+ART+ = HIV-positive participants on antiretroviral therapy; HIV+ART- = HIV-positive, antiretroviral therapy-naïve participants; HIV− = HIV-negative participants; M7 = 6 months post-dose 2; Pre = pre-vaccination; Y1 = 1 year post-dose 2; Y2 = 2 years post-dose 2; Y3 = 3 years post-dose 2.

Two doses of M72/AS01_E_ were immunogenic in all 3 cohorts, as previously reported^[17]^ and summarised in Figure 3. The immune response persisted up to 3 years post-vaccination. Seropositivity rates were 94.3%, 68.6%, and 97.1% at year 2 and 97.1%, 66.7%, and 97.3% at year 3 in the HIV+ART+, HIV+ART-, and HIV− cohorts, respectively.

**Figure 4:**
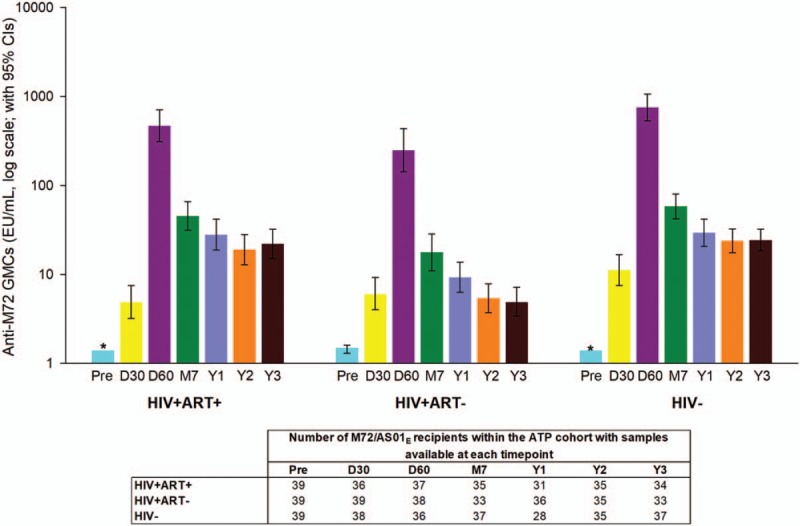
Geometric mean concentrations of antibodies against M72. Geometric mean concentrations are presented for participants who received M72/AS01_E_ and were included in the ATP cohort for immunogenicity. The colours represent the corresponding time points in Figure 5, 6, and 7. CI = confidence interval; D30 = 30 days post-dose 1; D60 = 30 days post-dose 2; EU = enzyme-linked immunosorbent assay units; GMCs = geometric mean concentrations; HIV+ART+ = HIV-positive participants on antiretroviral therapy; HIV+ART- = HIV-positive, antiretroviral therapy-naïve participants; HIV- = HIV-negative participants; M7 = 6 months post-dose 2; Y1 = 1 year post-dose 2; Pre = pre-vaccination; Y2 = 2 years post-dose 2; Y3 = 3 years post-dose 2. Note: ^∗^ The lower limit of the 95% CI is zero.

There was no notable decrease in seropositivity rate or GMCs after year 1. Two years post-vaccination, anti-M72 GMCs decreased to 19.1 EU/mL, 5.4 EU/mL and 23.9 EU/mL in HIV+ART+, HIV+ART-, and HIV− cohorts, respectively, and remained at 22.0 EU/mL, 4.9 EU/mL and 24.3 EU/mL at year 3 (Fig. 4). GMCs at years 2 and 3 continued to be significantly higher compared to pre-vaccination (*P*<.001 for all cohorts).

Humoral immune responses to M72 observed after vaccination were consistently lowest in the HIV+ART- cohort, whilst being higher and comparable in the HIV+ART+ and HIV− cohorts.

### 3.4 T-cell-mediated responses to M72/AS01_E_

**Figure 5:**
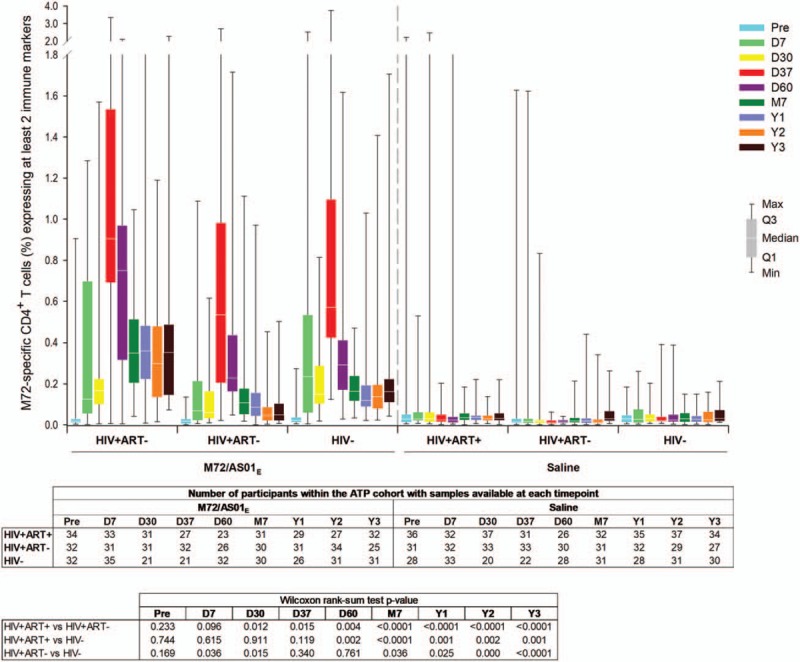
M72-specific CD4^+^ T-cell responses following vaccination with M72/AS01_E_. Data for all participants vaccinated with M72/AS01_E_ and included in the ATP cohort are presented as percentages of M72-specific CD4^+^ T-cells expressing at least 2 immune markers among IFN-γ, IL-2, TNF-α, and CD40L of all CD4^+^ T-cells, along with first and third quartiles and minimum and maximum values measured. % = percentage; D7 = 7 days post-dose 1; D30 = 30 days post-dose 1; D37 = 7 days post-dose 2; D60 = 30 days post-dose 2; HIV+ART+ = HIV-positive participants on antiretroviral therapy; HIV+ART- = HIV-positive, antiretroviral therapy-naïve participants; HIV− = HIV-negative participants; M7 = 6 months post-dose 2; Pre = pre-vaccination; Q1 = first quartile; Q3 = third quartile; Y1 = 1 year post-dose 2; Y2 = 2 years post-dose 2; Y3 = 3 years post-dose 2.

In participants who received M72/AS01_E_, M72-specific CD4^+^ T-cells expressing at least 2 immune markers among IL-2, IFN-γ, TNF-α, and CD40L peaked at 7 days post-dose 2.^[17]^ After this timepoint, the values gradually dropped and remained relatively constant from M7 up to year 3 post-vaccination (0.35%, 0.05%, and 0.15% of total CD4^+^ T-cells in the HIV+ART+, HIV+ART-, and HIV− cohorts, respectively). The overall amplitude of CD4^+^T-cell response was lowest in the HIV+ART- cohort (Fig. 5). In all cohorts, mainly quadruple-positive T-cell subsets were induced, up to year 3 post-vaccination (Fig. 6 ).

**Figure 6:**
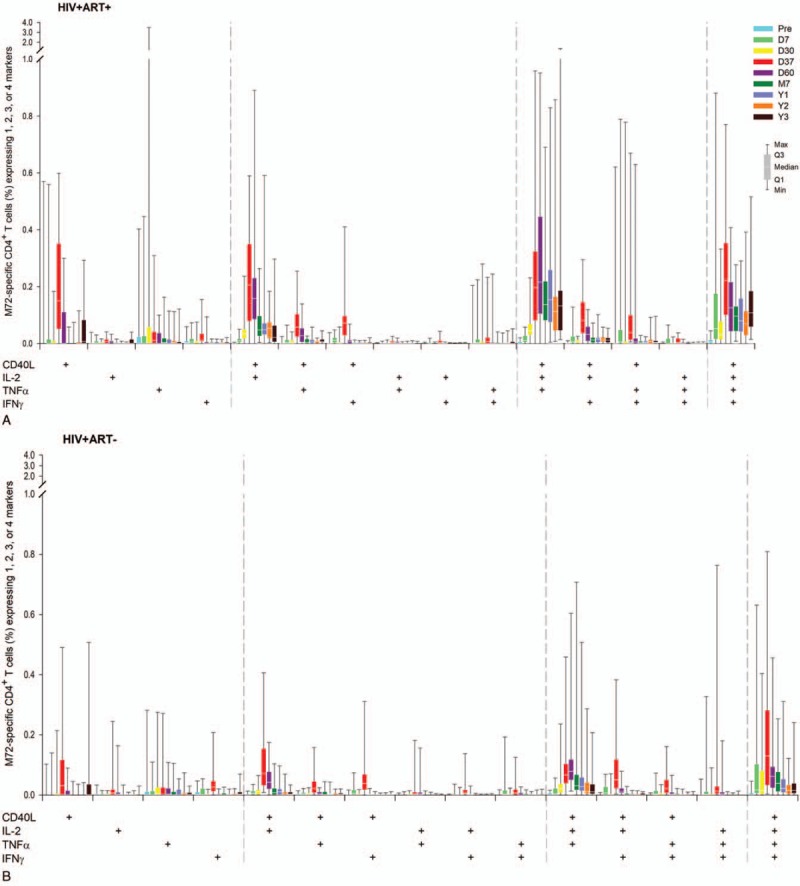
Immune-marker expression profiles following vaccination with M72/AS01_E_. Data for all participants vaccinated with M72/AS01_E_ and included in the ATP cohort are presented as percentages of M72-specific CD4^+^ T-cells expressing single immune markers among IFN-γ, IL-2, TNF-α and CD40L of all CD4^+^ T-cells and any combination of the 4 markers, along with first and third quartiles and minimum and maximum values measured. % = percentage; D7 = 7 days post-dose 1; D30 = 30 days post-dose 1; D37 = 7 days post-dose 2; D60 = 30 days post-dose 2; HIV+ART+ = HIV-positive participants on antiretroviral therapy; HIV+ART- = HIV-positive, antiretroviral therapy-naïve participants; HIV− = HIV-negative participants; M7 = 6 months post-dose 2; Pre = pre-vaccination; Q1 = first quartile; Q3 = third quartile; Y1 = 1 year post-dose 2; Y2 = 2 years post-dose 2; Y3 = 3 years post-dose 2.

**Figure 6 (Continued):**
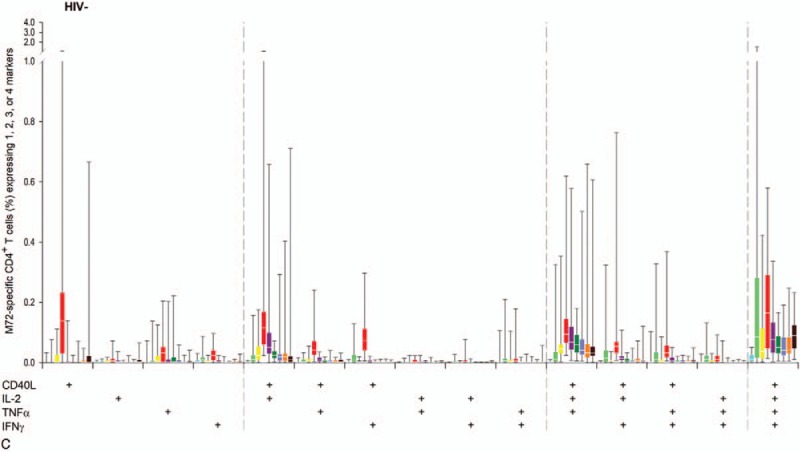
Immune-marker expression profiles following vaccination with M72/AS01_E_. Data for all participants vaccinated with M72/AS01_E_ and included in the ATP cohort are presented as percentages of M72-specific CD4^+^ T-cells expressing single immune markers among IFN-γ, IL-2, TNF-α and CD40L of all CD4^+^ T-cells and any combination of the 4 markers, along with first and third quartiles and minimum and maximum values measured. % = percentage; D7 = 7 days post-dose 1; D30 = 30 days post-dose 1; D37 = 7 days post-dose 2; D60 = 30 days post-dose 2; HIV+ART+ = HIV-positive participants on antiretroviral therapy; HIV+ART- = HIV-positive, antiretroviral therapy-naïve participants; HIV− = HIV-negative participants; M7 = 6 months post-dose 2; Pre = pre-vaccination; Q1 = first quartile; Q3 = third quartile; Y1 = 1 year post-dose 2; Y2 = 2 years post-dose 2; Y3 = 3 years post-dose 2.

No CD8^+^ T-cell response was observed following vaccination, as previously reported.^[17]^

**Figure 7:**
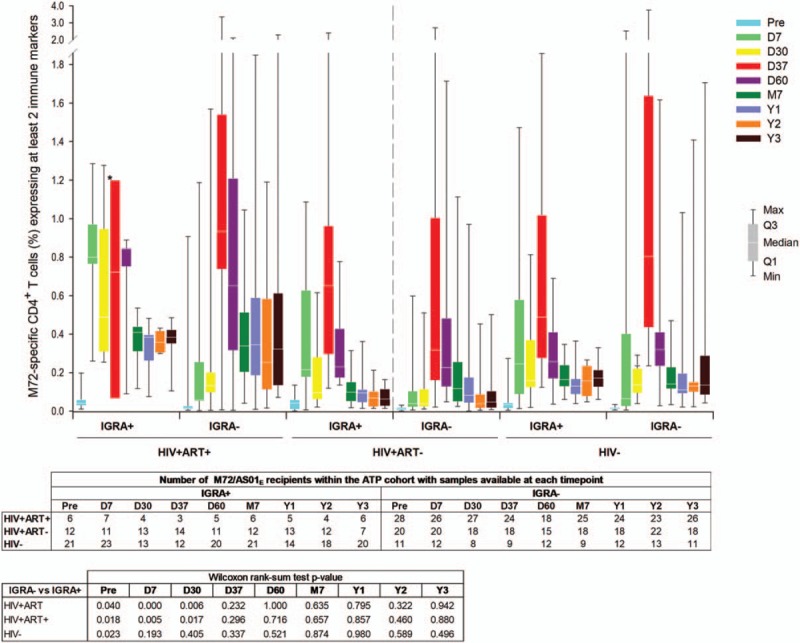
M72-specific CD4^+^ T-cell responses following vaccination with M72/AS01_E_ according to Interferon Gamma Release Assay results at baseline. Data for IGRA-positive and IGRA-negative participants vaccinated with M72/AS01_E_ and included in the ATP cohort are presented as percentages of M72-specific CD4^+^ T-cells expressing at least 2 immune markers among IFN-γ, IL-2, TNF-α, and CD40L of all CD4^+^ T-cells, along with first and third quartiles and minimum and maximum values measured. % = percentage; ATP = according-to-protocol; D7 = 7 days post-dose 1; D30 = 30 days post-dose 1; D37 = 7 days post-dose 2; D60 = 30 days post-dose 2; HIV+ART+ = HIV-positive participants on antiretroviral therapy; HIV+ART- = HIV-positive, antiretroviral therapy-naïve participants; HIV− = HIV-negative participants; IGRA = interferon-gamma release assay; M7 = 6 months post-dose 2; Pre = pre-vaccination; Q1 = first quartile; Q3 = third quartile; Y1 = 1 year post-dose 2; Y2 = 2 years post-dose 2; Y3 = 3 years post-dose 2.

In the HIV+ cohorts, the frequency of CD4^+^ T-cells expressing at least 2 immune markers among IL-2, IFN-γ, TNF-α, and CD40L was higher in IGRA-positive participants after the first vaccine dose (at D7 and D30, *P*≤.01), but these differences disappeared post-dose 2 (Fig. 7). There was no significant difference between IGRA sub-groups post-dose 1 and 2 among HIV− participants.

## 4 Discussion

During this long-term follow-up study, no SAEs related to the M72/AS01_E_ vaccine were reported and no safety concerns were raised. This suggests that the study vaccine was well tolerated in this population of ART-stable and ART-naïve HIV+ adults, as well as in HIV− adults.

M72/AS01_E_ was previously shown to induce a robust short-term immune response in HIV+ participants, irrespective of their ART status.^[17]^ The long-term immunogenicity data presented here confirm that albeit at lower values, M72-specific antibodies and polyfunctional CD4^+^ T-cells persist for up to 3 years post-vaccination. Persistence of immune responses for up to 3 years was first demonstrated in HIV− adults after 2 doses of a previous formulation of M72/AS01, which contained 40 μg of M72 and the adjuvant AS01_B_.^[10]^ Although the vaccine composition was not identical to the 1 used in this study, the immunogenicity profile of the 2 formulations was found to be comparable.^[11]^ Persistent antigen-specific polyfunctional CD4^+^ T-cell responses indicate the presence of circulating memory T-cells and have been associated with increased protection against other intracellular pathogens such as *Leishmania major* and HIV,^[18,19]^ as well as against *M tuberculosis*, in animal studies.^[20]^

Compared to HIV+ ART-naïve participants, participants receiving ART had a higher CD4^+^ T-cell response at years 2 and 3, with a similar magnitude as that observed in HIV− participants. This positive effect of ART on cellular immune responses is not a singular finding. A predominantly polyfunctional CD4^+^ T-cell response following 2 doses of M72/AS01_E_ was also observed in ART-stable participants in a previous study conducted in Switzerland,^[7]^ although persistence was only assessed for 6 months in that study. Also, in a long-term study with the MVA85A vector-based TB candidate vaccine, antigen-specific T-cell responses persisted for 6 years after vaccination in ART-stable participants, but waned after 3 to 5 years in ART-naïve participants.^[21]^

No significant difference between M72-specific CD4^+^ T-cell responses was observed after the second dose and until year 3, irrespective of the participants’ IGRA status at baseline, suggesting that pre-existent priming against *M tuberculosis* does not influence the cellular immune response elicited by M72/AS01_E_. This is in line with previous observations from a phase II clinical trial with M72/AS01_E_ in HIV− adolescents.^[12]^ In all cohorts in our study, at years 2 and 3, the magnitude of CD4+ T cell responses and the degree of polyfunctionality of CD4+ T cells were unchanged from those observed at year 1 after the second dose.^[17]^

There is evidence that an Mtb-specific antibody response could be important in protecting against TB,^[22,23]^ although the role of vaccine-induced antibodies remains to be established. Preservation of naïve T-cell populations could impact antigen-specific antibody responses, as observed for influenza vaccines,^[24]^ therefore initiation of ART therapy is likely to enhance humoral responses as well. Our results also show a positive influence of ART on the persistence of anti-M72 IgG response, which was higher in the HIV+ ART-stable than in the HIV+ART-naïve participants.

This study was designed to assess primarily the safety and reactogenicity of M72/AS01_E_ vaccine in HIV+ adults. To confirm the observed differences in immune responses between HIV+ART+ and HIV+ART- cohorts, specifically designed studies would be needed. The current World Health Organization (WHO) recommendation is to treat all HIV+ persons, irrespective of their CD4^+^ T-cell count level. Therefore, this study carried out at the time when the WHO recommended a cut-off of ≤350 CD4^+^ T-cells/mm^3^ for ART initiation, is relevant for the assessment of M72/AS01_E_ in HIV+ ART-naïve persons. Other limitations of this study include the fact that the clinical relevance of the immunogenicity data presented here is difficult to establish in the absence of a correlate of protection. Efficacy trials would be needed to assess the protective effect of these persistent immune responses. Additionally, enrolment was not stratified or controlled for IGRA status; therefore the number of participants included in the IGRA-positive sub-group was low. Also, the study was not designed, nor powered, to make comparisons between cohorts. Finally, CMI responses were analysed taking into account only conventional T-cells, expressing a limited number of markers.

## 5 Conclusion

The candidate M72/AS01_E_ vaccine-induced humoral and cellular immune responses that persisted up to 3 years following vaccination in both HIV− and HIV+ adults, regardless of their ART status. No significant safety concerns were raised concerning further studies of this vaccine in HIV+ populations.

**Figure 8:**
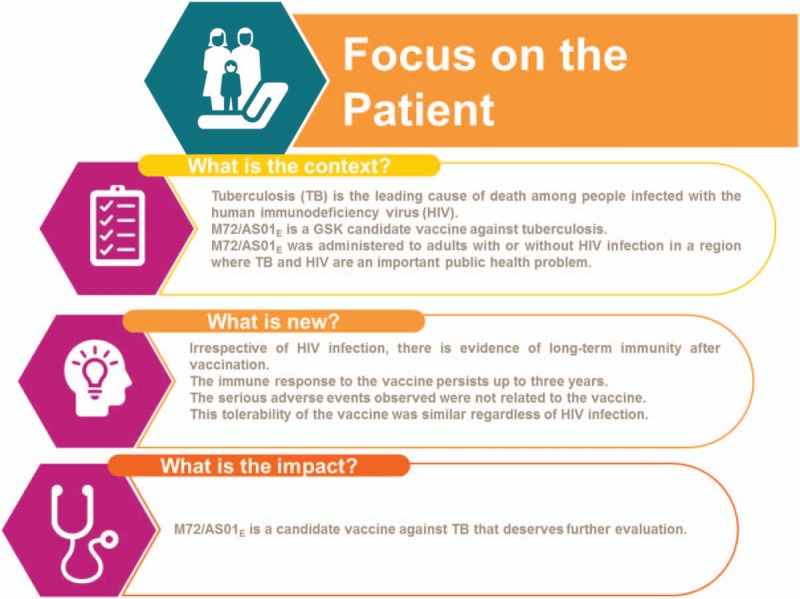
Focus on the patient section.

Figure 8 represents a “focus on the patient” section, which elaborates on the clinical relevance of the research intended to be shared with patients by health care professionals.

## Acknowledgments

We like to thank Ms. Pearl, regulatory coordinator and all the research nurses, counsellors, retention staff, pharmacists, QA/QC personnel, data team and the lab staff at the Clinical Research Site of YRGCARE Medical Centre, VHS, Chennai, India for their involvement in this clinical trials.

The authors thank Timea Kiss (XPE Pharma & Science for GSK Vaccines) for medical writing services, and William Zonta (XPE Pharma & Science for GSK Vaccines) for manuscript coordination.

All authors participated in the design, implementation or analysis, the interpretation of the study, and the development of this manuscript. All authors had full access to the data and gave final approval before submission. The corresponding author was responsible for submission of the publication.

## Author contributions

**Conceptualization:** Nagalingeswaran Kumarasamy, Leo Njock Ayuk, Anne Bollaerts, Marie-Ange Demoitié, Erik Jongert, Opokua Ofori-anyinam.

**Formal analysis:** Nagalingeswaran Kumarasamy, Selvamuthu Poongulali, Elaine Jacqueline Akite, Leo Njock Ayuk, Anne Bollaerts, Marie-Ange Demoitié, Erik Jongert, Opokua Ofori-anyinam, Olivier Van Der Meeren.

**Resources:** Nagalingeswaran Kumarasamy, Selvamuthu Poongulali, Faith Esther Beulah, Elaine Jacqueline Akite, Leo Njock Ayuk, Anne Bollaerts, Marie-Ange Demoitié, Erik Jongert, Opokua Ofori-anyinam, Olivier Van Der Meeren.

**Supervision:** Olivier Van Der Meeren, Opokua Ofori-anyinam, Nagalingeswaran Kumarasamy.

**Writing – original draft:** Elaine Jacqueline Akite, Olivier Van Der Meeren.

**Writing – review & editing:** Nagalingeswaran Kumarasamy, Selvamuthu Poongulali, Faith Esther Beulah, Elaine Jacqueline Akite, Leo Njock Ayuk, Anne Bollaerts, Marie-Ange Demoitié, Erik Jongert, Opokua Ofori-anyinam, Olivier Van Der Meeren.

Olivier Van Der Meeren orcid: 0000-0003-0291-743X.
