# Long-term safety and immunogenicity of the M72/AS01E candidate tuberculosis vaccine in HIV-positive and -negative Indian adults: Results from a phase II randomized controlled trial: Erratum

**DOI:** 10.1097/MD.0000000000013948

**Published:** 2018-12-28

In the article, “Long-term safety and immunogenicity of the M72/AS01E candidate tuberculosis vaccine in HIV-positive and -negative Indian adults: Results from a phase II randomized controlled trial”,^[1]^ which appeared in Volume 97, Issue 45 of *Medicine*, the disclosure statement should be “The authors report no other conflicts of interest.”

In Figure 5, the label for the first group should be HIV+ART+” instead of “HIV+ART−“.

**Figure:**
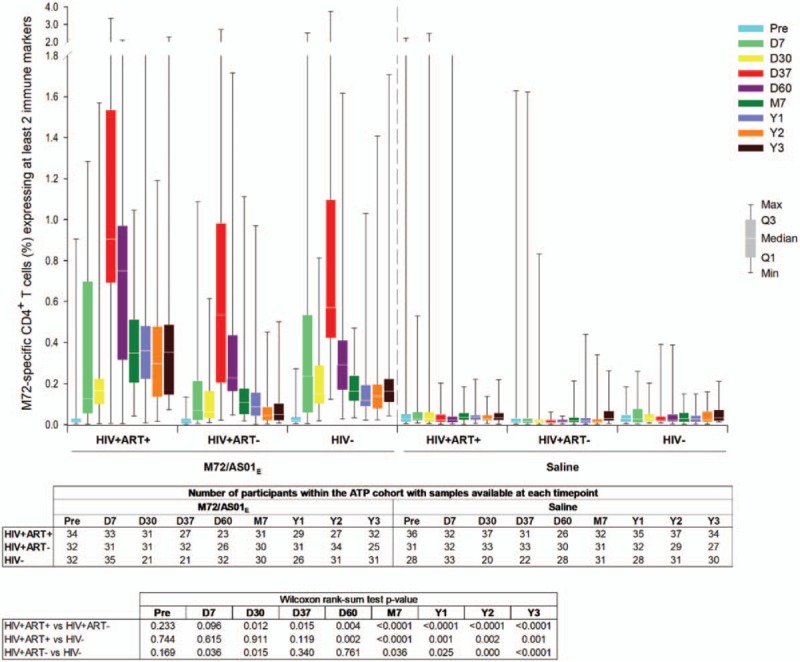


In the caption for Figure 6, “D6 = 30 days post-dose 2” should be “D60 = 30 days post-dose 2”.
